# Blockchain-Enabled Uncertainty-Aware Passive Wi-Fi Localization for Secure Critical Infrastructure Sensor Networks

**DOI:** 10.3390/s26092797

**Published:** 2026-04-30

**Authors:** Dmytro Prokopovych-Tkachenko, Oleksandr Galushchenko, Olga Torstensson, Volodymyr Zvieriev, Saltanat Adilzhanova, Edison Pignaton de Freitas

**Affiliations:** 1 Department of Cybersecurity and Information Technologies, University of Customs and Finance, 4900 Dnipro, Ukraine; omega2417@umsf.edu.ua; 2 Faculty of Management and Information Security, Vinnytsia National Technical University, 21021 Vinnytsia, Ukraine; 00-20-031.stud@vntu.edu.ua; 3 School of Information Technology, Halmstad University, 301 18 Halmstad, Sweden; olga.torstensson@hh.se; 4 Department of Software Engineering and Cybersecurity, State University of Trade and Economics, 02156 Kyiv, Ukraine; zvieriev_vp@knute.edu.ua; 5 Department of Computer Engineering, Faculty of Information Technology, Almaty Technological University, Almaty 050012, Kazakhstan; 6 Institute of Informatics, Federal University of Rio Grande do Sul, Porto Alegre 91501-97, Brazil

**Keywords:** wireless sensor networks, critical infrastructure security, localization, uncertainty, posterior distribution, calibration, decision thresholds, robustness, filtering, telemetry, blockchain-based evidentiary logging, permissioned blockchain, on-chain/off-chain architecture

## Abstract

**Highlights:**

**What are the main findings?**

- Passive Wi-Fi localization is more useful for SOC operations when reported as a posterior distribution with calibrated uncertainty rather than as a single coordinate estimate.
- Field experiments identify an operational boundary at about 40 m, beyond which localization errors increase markedly, and fully automated actions become less reliable.

**What are the implications of the main findings?**

- Calibrated confidence can be used to tune AUTO, VERIFY, and OBSERVE response modes.
- A permissioned blockchain layer can strengthen the integrity and auditability of localization evidence while keeping raw telemetry off chain, and practical deployment depends on stable antenna geometry, field calibration, and mobile or UAV-assisted data collection.

**Abstract:**

Passive Wi-Fi localization for critical-infrastructure security operations centers (SOCs) faces three interconnected limitations. First, many existing methods produce single-point coordinate estimates without calibrated uncertainty, making them unsuitable for automated SOC response. Second, localization pipelines often lack an evidentiary chain of custody, limiting reliable post-incident auditability. Third, SOC automation cannot safely rely on uncalibrated confidence values because erroneous high-impact actions and missed escalations carry asymmetric operational costs. This study presents a Blockchain-Enabled Uncertainty-Aware Passive Wi-Fi Localization framework for heterogeneous sensor networks composed of stationary sensors, mobile receivers, and UAV-assisted collection nodes. Instead of producing a single coordinate estimate, the method derives a posterior spatial distribution with calibrated uncertainty from monitor-mode observations, including RSSI aggregates, management/control frame features, channel occupancy indicators, and receiver logs. The framework combines three tightly coupled components: (i) Bayesian coordinate estimation with robust loss functions and range-dependent error modeling; (ii) uncertainty calibration that converts posterior confidence into operational SOC response modes (AUTO, VERIFY, and OBSERVE) via empirical coverage metrics and reliability diagrams; and (iii) a permissioned evidentiary logging layer that anchors integrity-relevant metadata and policy labels on-chain while keeping raw telemetry off-chain for tamper-evident auditability and scalability. The coupling between layers is explicit: calibrated confidence scores govern smart-contract gating conditions, and smart-contract policy thresholds feed back into the calibration stage. Field validation shows that localization performance degrades markedly beyond approximately 40 m, indicating a practical boundary for confident automated action. The proposed framework integrates passive sensing, uncertainty-aware localization, and blockchain-based evidentiary trust for secure critical-infrastructure sensor networks. Its key contributions are: (1) a posterior-distribution-based passive localization pipeline; (2) empirical coverage metrics for calibrating SOC response thresholds; (3) a hybrid on-chain/off-chain architecture linking localization outputs to a permissioned ledger; and (4) field validation establishing the 40 m operational validity boundary.

## 1. Introduction

Wi-Fi infrastructure in critical environments serves not only legitimate communications but can also become a source of radio activity associated with reconnaissance, misuse, or attack preparation [1]. For a security operations center (SOC), the relevant question is not only where the source is likely to be, but also how reliable that estimate is. In this setting, localization errors have asymmetric costs: an incorrect response may disrupt operations, whereas a missed event may permit further escalation. SOC-oriented localization must therefore report uncertainty explicitly rather than rely on a single coordinate estimate.

In critical-infrastructure settings, localization outputs function not only as technical estimates but also as operational evidence. They may support incident triage, physical verification, access-control escalation, or automated SOAR playbooks. This introduces an evidentiary requirement: the information used to justify a response should remain trustworthy, auditable, and resistant to post hoc modification. Blockchain technology is relevant here because it provides a tamper-evident ledger in which records are linked by cryptographic hashes, making unauthorized modification detectable [2]. In permissioned blockchain systems, participation is restricted to vetted entities, which is more appropriate for critical-infrastructure environments because it preserves confidentiality while retaining auditability and non-repudiation [2,3]. These requirements are also consistent with cybersecurity log-management planning guidance [4]. Smart contracts further allow policy conditions, such as minimum calibrated confidence or evidence completeness, to be encoded in an auditable form [5].

For passive Wi-Fi localization, a hybrid on-chain/off-chain design is particularly appropriate. Raw packet captures, receiver logs, and derived feature vectors are often too voluminous and sensitive for direct on-chain storage. By contrast, compact decision artifacts such as hashes, timestamps, posterior summaries, confidence values, and policy labels can be anchored on a permissioned ledger. In this role, blockchain does not improve localization accuracy itself; rather, it strengthens integrity, provenance, and the auditability of localization-derived decisions [3,6].

Despite this potential, several research problems arise when blockchain is integrated with passive Wi-Fi localization. First, passive monitor-mode localization relies on noisy RSSI and management/control-frame features that are affected by multipath propagation and environmental variability, so calibrated uncertainty must accompany each evidentiary record. Second, localization outputs are probabilistic, whereas smart-contract policies are deterministic and threshold-based, requiring a principled mapping from posterior uncertainty to operational gating decisions. Third, real-time telemetry generation conflicts with the latency and storage cost of on-chain recording, requiring selective anchoring to preserve evidentiary value while remaining scalable. Fourth, MAC randomization and intermittent transmitter activity complicate persistent source tracking and therefore the logging of state transitions. Finally, the coupling between the localization layer and the blockchain layer is bidirectional: policy thresholds influence calibration targets, while calibrated localization outputs determine whether automated action is permitted.

The research gap lies not in the lack of localization methods, but in the absence of an SOC-oriented formulation that simultaneously satisfies six requirements: (a) the output is a posterior coordinate distribution with confidence regions, rather than a point estimate; (b) uncertainty is calibrated and translated into operational decision thresholds; (c) robust mechanisms mitigate outliers and interference; (d) temporal dynamics of intermittent and mobile sources are handled; (e) validation is performed under field conditions using mobile and unmanned data collection; and (f) localization evidence is protected against post-collection modification through tamper-evident evidentiary logging. To the best of our knowledge, no prior work combines calibrated Bayesian uncertainty estimation, SOC-oriented decision gating, and permissioned blockchain-based evidentiary logging in a single passive Wi-Fi framework.

The contributions of this study are as follows:

1. A three-stage passive localization pipeline that produces posterior spatial distributions with calibrated uncertainty from monitor-mode Wi-Fi observations, without requiring active client participation.
2. Empirical coverage metrics and reliability diagrams for translating posterior uncertainty into verifiable SOC response thresholds (AUTO, VERIFY, OBSERVE).
3. A hybrid on-chain/off-chain architecture that couples localization confidence scores to smart-contract gating conditions, providing tamper-evident evidentiary logging while preserving telemetry confidentiality.
4. Field validation across two range regimes, demonstrating a practical 40 m operational validity boundary and explicitly characterizing the localization error spike beyond that range.

Section 2 reviews related work. Section 3 formalizes the methodology. Section 4, Section 5 and Section 6 describe the mathematical model, algorithmic implementation, and system architecture. Section 7, Section 8, Section 9 and Section 10 present the experimental results, discussion, and conclusions.

## 2. Related Work

Wi-Fi localization research spans several methodological families, each with distinct trade-offs in accuracy, infrastructure requirements, and suitability for passive SOC deployments.

RSSI-based and geometric methods. Traditional approaches rely on RSSI measurements and geometric models (trilateration, weighted centroid) to estimate source coordinates [7]. These methods produce point estimates without associated uncertainty and are sensitive to multipath propagation, shielding, and environmental variability [8], making them operationally unsafe for automated SOC response.

AI-based localization. Machine learning and fingerprinting approaches improve nominal accuracy by learning the mapping from radio features to coordinates using k-NN, neural networks, or Gaussian processes [7]. AI-based location tracking in Wi-Fi indoor positioning achieves sub-meter accuracy under controlled conditions. However, these methods require extensive calibration databases, suffer from domain shift, and still output point estimates. Uncertainty prediction for fingerprinting has been explored [9], but integration with SOC decision logic and evidentiary pipelines remains an open problem.

Networking-function-based and passive sensing approaches. Physical-layer jammer detection in multi-hop IoT networks and management-frame-based intrusion detection [10,11] demonstrate that passive monitor-mode observations carry rich spatial and behavioral information. Passive probing and reuse of Wi-Fi signaling as a sensing channel are active research directions [12], and UAV-based radio environment mapping extends passive spatial coverage [13]. These approaches suit SOC deployments but do not produce calibrated uncertainty estimates.

Robustness and uncertainty calibration. Robust loss functions and M-estimation reduce outlier influence [14,15]. Calibration of probabilistic predictions via reliability diagrams and coverage metrics is described in [16,17]; applying such calibration to localization-derived confidence scores for SOC threshold control is a key contribution of the present work.

Blockchain-based evidentiary systems. Permissioned blockchain architectures have been applied to auditable logging for cybersecurity artifacts [2], chain-of-custody for physical evidence [2], and AI-blockchain integration for real-time security workflows [6]. Smart contracts support automated cybersecurity compliance and threat response [5]. However, these systems have not previously been coupled to probabilistic localization pipelines where the confidence score directly governs on-chain policy enforcement.

Transport-layer security context. Sensor networks in critical infrastructure transmit telemetry over standard protocols including TCP and QUIC. Comparative analysis of QUIC and TCP in unsafe networks [18] shows that transport-layer integrity alone is insufficient for evidentiary purposes: an attacker with analytics-layer access can modify events after they cross the transport layer. This motivates the on-chain hash-anchoring approach adopted in this work.

The present work integrates passive monitor-mode sensing, Bayesian uncertainty-aware localization, and permissioned evidentiary logging into a single SOC-oriented pipeline. This combination has not been addressed in the reviewed literature.

## 3. Methodology

The proposed method is formulated as a passive localization pipeline for security operations centers (SOCs). Instead of producing a single coordinate, it estimates a posterior spatial distribution with associated uncertainty, which is then used to define response thresholds and audit conditions within SIEM/SOAR workflows.

All inputs are collected passively in monitor mode and include RSSI aggregates, temporal and frequency characteristics of management and control frames, channel occupancy indicators, and receiver logs. Active ranging methods such as IEEE 802.11mc FTM/RTT are excluded because they require client participation and therefore fall outside the passive monitoring assumptions of this study. This formulation reflects SOC practice, in which automated actions should be triggered only when localization confidence is sufficiently reliable and properly calibrated [19,20].

**Figure 1:**
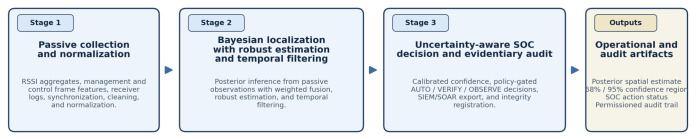
Three-stage passive Wi-Fi localization pipeline.

The methodology comprises three stages, as illustrated in Figure 1. The first stage performs passive data acquisition, cleaning, synchronization, and normalization. This includes robust aggregation of RSSI values within temporal windows, transformation of management and control frames into stable descriptive features, and alignment of receiver logs for subsequent fusion. The second stage performs Bayesian coordinate estimation by constructing likelihood functions for passive observations and deriving posterior distributions with associated confidence regions. Robust estimation methods, including M-estimation and Huber-type loss functions, are used to reduce the effect of outliers and interference in complex radio environments [14,15]. The third stage calibrates posterior uncertainty and converts it into operational decision thresholds. Empirical coverage metrics and reliability diagrams are used to determine whether an event should trigger automatic action, require verification, or remain under observation [16,17]. The resulting estimates are exported to SIEM as structured events containing uncertainty fields and evidentiary telemetry [19].

Because localization quality in field conditions depends strongly on observation geometry, the method also incorporates mobile and UAV-assisted data collection. Controlled trajectories and sampling at approximately 1 Hz improve spatial coverage, support statistical accumulation, and increase reproducibility under realistic operating conditions [13,21].

To support auditability, the pipeline is extended with a permissioned evidentiary logging layer. Each localization result is represented by two linked artifacts: an operational event delivered to SIEM/SOAR and an evidentiary record registered in a permissioned ledger. The ledger stores hashes, timestamps, event and model identifiers, confidence summaries, policy labels, and references to protected off-chain evidence containers, whereas raw packet captures, receiver logs, and derived feature vectors remain off chain for confidentiality and scalability [2,3,6]. Smart contracts are used only for policy enforcement. They verify whether predefined conditions for automated action are satisfied, such as sufficient calibrated confidence, acceptable confidence-region size, evidence completeness, and successful integrity registration. If these conditions are not met, the system assigns a VERIFY or OBSERVE status instead of authorizing automatic action [5].

The following subsections describe the mathematical formulation of the likelihood and posterior models, the robustness and temporal filtering mechanisms, and the rules used to convert calibrated uncertainty into SOC decision thresholds.

## 4. Mathematical Model

### 4.1. Posterior Spatial Distribution and Likelihood Model for Passive Wi-Fi Observations

This section defines the posterior distribution $p\left(x|z\right)$ over source coordinates x ∈ ℝ^2^ given passive observations z, and the likelihood $p\left(z|x\right)$ derived from monitor-mode RSSI statistics and frame features. Intuitively, the posterior encodes the probability that the unknown source is located at each candidate position x, updated from the prior p(x) by the evidence in z.

Let x ∈ ℝ^2^ denote the unknown coordinates of the Wi-Fi source and z = {zi}_i=1_^N^ the set of passive observations collected by N sensors. The Bayesian posterior is:

(1) $$p\left(x|z\right)\propto p\left(z|x\right)p(x)$$

where ∝ denotes proportionality up to a normalizing constant (i.e., p(x|z) = p(z|x)p(x)/p(z), where p(z) = ∫p(z|x)p(x)dx is the evidence). p(x) is the prior distribution—for example, uniform within the controlled area with a mask for physically inaccessible regions. p(z|x) is the likelihood, representing how probable the observed measurements are if the source were at position x.

Assuming conditional independence of sensor observations given the source location:

(2) $$p\left(z|x\right)=\;\prod_{i=1}^{N}p(z_{i}|x)$$

This factorization holds when measurement errors across sensors are uncorrelated given *x*, although this assumption is relaxed in practice by the robust weighting scheme in Section 4.3.

### 4.2. Passive RSSI-Based Measurement Model

Let the aggregated RSSI measurement be denoted as $r_{i}$, for example, as the median or a robust mean computed over a time window. The passive measurement model is defined as:

(3) $$r_{i}=\mu_{i}(d_{i}(x),\theta_{i})+\varepsilon_{i},$$

where $d_{i}(x)=∥x-s_{i}{∥}_{2}\;$ is the Euclidean distance between the candidate source position x and sensor i, located at $s_{i}$; $\mu_{i}(\cdot )$ is a monotonic signal-attenuation function parameterized by environmental and channel parameters $\theta_{i}$; and $\varepsilon_{i}$ is the error term that captures interference and multipath propagation.

For Bayesian estimation, $\varepsilon_{i}$ is modelled as a zero-mean measurement error with distance-dependent variance, as further specified in Section 4.5. This formulation provides the baseline probabilistic measurement model used in the likelihood function, while the robust loss introduced in Section 4.4 reduces the influence of non-Gaussian outliers caused by multipath propagation, interference, and transient channel disturbances.

### 4.3. Weighted Sensor Fusion

Considering heterogeneity among sensors, weights are introduced:

(4) $$-\log p\left(z|x\right)=\sum_{i=1}^{N}w_{i}l_{i}(x)$$

where $l_{i}(x)$ is the local negative log-likelihood and the weights satisfy:

$$w_{i}\geq 0,\;\;\sum_{i}w_{i}=1\;$$

This formulation assigns greater influence to sensors whose observations are more reliable under the current channel and geometry conditions, while reducing the impact of noisy or unstable sensors. As a result, the fused posterior is less sensitive to local anomalies and better reflects the quality of the available passive observations.

A practical weighting form:

(5) $$w_{i}\propto \;k_{i}/((1+{occ}_{i})(1+\alpha_{i,noise}^{2}))$$

### 4.4. Robust Loss

To reduce the influence of outliers, a robust loss function (e.g., Huber loss) is applied:

(6) ρδu=12u2, |u|≤δδu−12δ, |u|>δ

with normalized residual:

$$u=\left(r_{i}-\mu_{i}\left(d_{i}\left(x\right),\theta_{i}\right)\right)/\sigma (d_{i})$$

The robust loss limits the influence of large residuals that may arise from interference, bursty management-frame activity, or transient channel disturbances. In practical terms, this prevents a small number of corrupted observations from disproportionately shifting the posterior estimate.

### 4.5. Range-Dependent Uncertainty with Explicit Spike Beyond ≈40 m

To avoid overestimating localization accuracy, the distance-dependent variance is modeled with an explicit spike beyond d_0_ ≈ 40 m:

(7) $$\sigma^{2}\left(d\right)=\sigma_{0}^{2}\left(1+\alpha d^{2}\right)+\beta \cdot spike(d;d_{0},∆)$$

where

(8) $$spike\left(d;d_{0},∆\right)=({1+e^{-(d-d_{0})/∆})}^{-1}$$

Here, Δ denotes the additional variance increment introduced beyond the operational distance threshold *d*0.

Physically, it represents the abrupt increase in localization uncertainty caused by non-line-of-sight propagation, attenuation variability, local reflections, and interference in the far-range regime. Operationally, this term prevents the model from overstating confidence beyond approximately 40 m.

### 4.6. Confidence Region

(Ellipse/Credible Set)

Assuming

$$p\left(x|z\right)\approx N(\mu ,\Sigma )$$

the confidence region at level α is:

(9) $$R_{\alpha }=\left\{x:{\left(x-\mu \right)}^{T}\Sigma^{-1}(x-\mu )\leq \chi_{2,\alpha }^{2}\right\}$$

This confidence region defines the spatial area within which the source is expected to lie with probability level α under the posterior model. In the SOC setting, the size and placement of this region are operationally important because they determine whether the estimate is sufficiently precise to justify automatic action or whether verification is required.

### 4.7. Temporal Filtering (Intermittent/Moving Sources)

Temporal filtering stabilizes the localization output for intermittent or moving transmitters by combining current observations with the previous posterior state. This is particularly important in operational monitoring environments, where short bursts of activity, packet loss, or transient interference can otherwise produce unstable coordinate estimates.

For moving or intermittent transmitters, recursive Bayesian filtering is applied:

(10) $$p\left(x_{t}|z_{1:t}\right)\propto p(z_{t}|x_{t})\int p\left(x_{t}|x_{t-1}\right)p(x_{t-1}|z_{1:t-1}){dx}_{t-1}$$

### 4.8. ROC and CDF Definitions

Localization error:

(11) $$e={\left\|x-x^{*}\right\|}_{2}$$

Error cumulative distribution function:

(12) $$F_{E(r)}=Pr(e\leq r)$$

### 4.9. Mobile Collection Statistical Accumulation Model

Let a mobile platform collect measurements with frequency λ (e.g., 1 Hz).

Under the assumption of weakly correlated measurement errors, the posterior covariance decreases with the number of measurements M:

(13) $$\sum \left(M\right)\approx (1/M_{eff})\Sigma_{1}$$

where $M_{eff}\leq M$ accounts for correlation caused by channel hopping, multipath propagation, and repeated trajectory segments.

## 5. Algorithmic Implementation

Only the general framework is presented here, without disclosing implementation details that would reveal internal know-how.

General Framework of Passive Bayesian Localization with SOC Thresholds.

The proposed procedure is summarized in Algorithm 1. The algorithm is presented as an array to improve readability and to clearly show the sequence of passive data collection, Bayesian posterior updating, uncertainty calibration, and SOC decision assignment.
**Algorithm 1.** Passive Bayesian Localization with SOC Thresholds.$\mathrm{Inputs}:\;\mathrm{sensor}\;\mathrm{coordinates}\;s_{i}$$;\;\mathrm{passive}\;\mathrm{observations}\;z_{1:T}$; prior p(x); parameters of range-dependent uncertainty; $\mathrm{SOC}\;\mathrm{thresholds}\;\tau_{auto}$$\;\mathrm{and}\;\tau_{verify}$.$\mathrm{Outputs}:\;\mathrm{posterior}\;p(x_{t}|z_{1:t})$$;\;\mathrm{estimated}\;\mathrm{coordinates}\;x^{t}$$;\;\mathrm{confidence}\;\mathrm{region}\;R_{\alpha ,t}$$;\;\mathrm{SOC}\;\mathrm{decision}\;D_{t}$.$\mathrm{Initialize}\;p(x_{0})$ and calibration parameters (obtained from field experiments).For t = 1 … T:Collect passive features: RSSI statistics, management/control frame features, and receiver logs.$\mathrm{Estimate}\;\mathrm{sensor}\;\mathrm{weights}\;w_{i}$ (reliability, channel occupancy, stability).$\mathrm{Construct}\;\mathrm{likelihood}\;p(z_{t}|x_{t})$ using robust loss and σ^2^(d) with an error spike beyond ≈ 40 m.$\mathrm{Update}\;\mathrm{posterior}\;p(x_{t}|z_{1:t})$ (with temporal prediction if required).$\mathrm{Compute}\;\mathrm{coordinate}\;\mathrm{estimate}\;x^{t}$$\;\mathrm{and}\;\mathrm{confidence}\;\mathrm{region}\;R_{\alpha ,t}$.$\mathrm{Evaluate}\;\mathrm{calibrated}\;\mathrm{confidence}\;C_{t}$ (coverage/reliability).$\mathrm{Determine}\;\mathrm{SOC}\;\mathrm{decision}:\;\mathrm{if}\;C_{t}\geq \tau_{auto}\to D_{t}=AUTO$$;\;\mathrm{if}\;C_{t}\geq \tau_{verify}\to D_{t}=VERIFY$$;\;\mathrm{otherwise}\;D_{t}=OBSERVE$.

Export event to SIEM including uncertainty fields and evidentiary telemetry.

### 5.1. Complexity Analysis

Let the posterior be approximated on a grid of G candidate points or particles. The likelihood computation per step has complexity O(N·G), with an additional O(G) for normalization and (if required) prediction. In practice, G is adapted according to the expected uncertainty (coarse-to-fine strategy).

### 5.2. Reproducibility (General Framework)

Reproducibility is ensured at the level of the general framework through the specification of data formats, collection parameters (frequency, channels), field experiment protocols, evaluation metrics, and calibration principles. Details of internal correlation and processing procedures are intentionally not disclosed.

## 6. System Architecture

**Figure 2:**
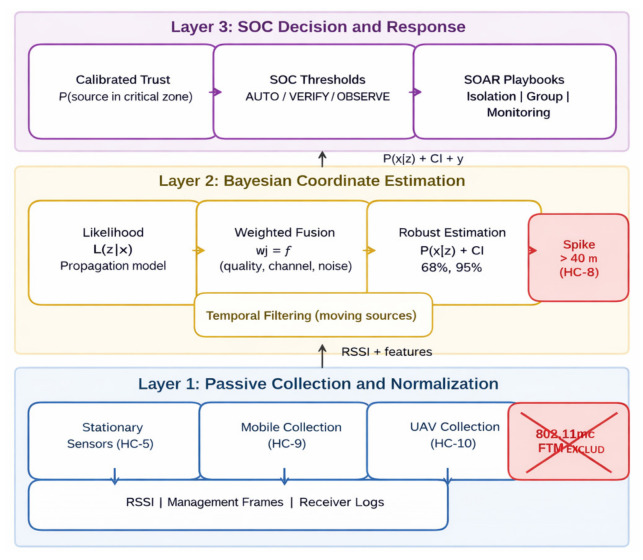
Multi-layer architecture of spatial attribution of Wi-Fi activity in critical infrastructure.

The system architecture is structured around three design principles that directly follow from the SOC requirements established in Section 2. First, technological separation is enforced: passive radio data (RSSI, management/control frames, and receiver logs) are kept separate from protected network events and SOC response logic, with correlation restricted to monitor-mode observations and security logs. This separation is necessary because merging raw telemetry with protected network identifiers at the collection layer would undermine both privacy and data-minimization requirements. Second, the output of the localization pipeline is probabilistic: the architecture propagates a posterior distribution and its associated uncertainty through all downstream layers so that SOC decisions are grounded in calibrated confidence rather than point estimates. Third, the evidentiary layer is decoupled from the localization layer: the blockchain anchors integrity proofs and policy-transition records but does not alter or replace the probabilistic inference, allowing the two layers to be updated, audited, or replaced independently. Figure 2 shows the resulting three-layer architecture. Layer 1 collects and normalizes passive data from stationary sensors, mobile platforms, and UAV sources. Layer 2 performs Bayesian fusion (likelihood, robust weighting, and posterior estimation) and generates confidence regions while accounting for the distance-related error spike beyond approximately 40 m. Layer 3 converts calibrated uncertainty into SOC decision thresholds (AUTO, VERIFY, and OBSERVE) and triggers SOAR playbooks.

Passive sources include RSSI, management frames, control frames, and logs, with correlation only to protected network events. IEEE 802.11mc FTM/RTT is classified as active-only and excluded from passive monitoring. Layer 1 collects and normalizes passive data from stationary sensors, mobile platforms, and UAV sources. Layer 2 performs Bayesian fusion (likelihood → weighting → robust estimation) to produce the posterior distribution and confidence regions while accounting for the distance-related error spike beyond ≈ 40 m. Layer 3 converts uncertainty into calibrated confidence and SOC decision thresholds (AUTO/VERIFY/OBSERVE) and triggers SOAR playbooks.

**Figure 3:**
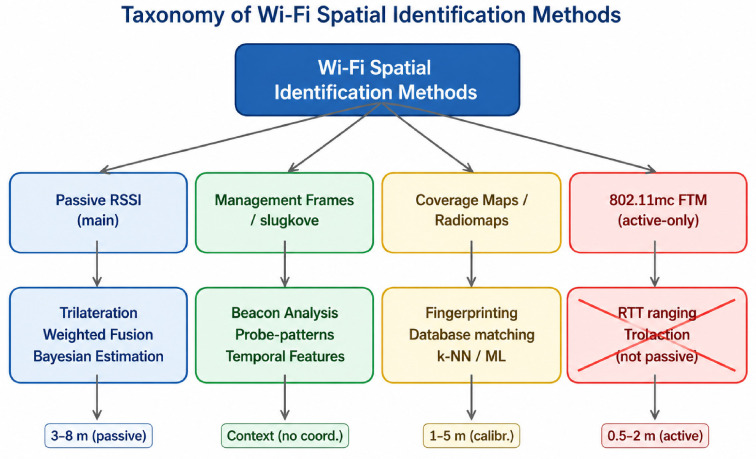
Taxonomy of Wi-Fi localization methods: passive approaches (RSSI, management/control frames, coverage maps/radio maps, reference measurement databases) and active-only approaches (IEEE 802.11mc FTM/RTT). No prohibited terminology is used.

Figure 3 presents a taxonomy of Wi-Fi spatial identification methods:

(1) Passive RSSI-based methods as the primary data channel (trilateration/weighted fusion/Bayesian estimation, typically ~3–8 m in passive mode);
(2) Management/control frame analysis (beacon analysis, probe patterns, temporal features—primarily providing contextual information rather than precise coordinates);
(3) Coverage maps/radio maps and fingerprinting (matching with a reference measurement database using k-NN or machine learning methods, typically ~1–5 m when calibration is available);
(4) IEEE 802.11mc FTM as an active-only approach (RTT ranging/trilateration, ~0.5–2 m), which in this work is explicitly marked as unsuitable for passive SOC monitoring and therefore excluded.

Next, the practical pipeline is presented through which the SOC obtains passive Wi-Fi localization without active measurements, with correlation strictly limited to events from the protected network.

**Figure 4:**
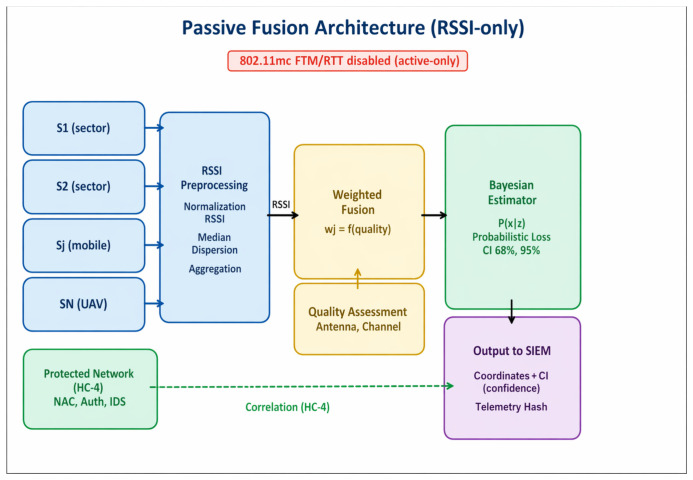
Multi-source passive fusion scheme (RSSI-only as the basis for localization) with allowed correlation only between monitor-mode observations and protected network data.

Figure 4 illustrates the architecture of multi-source passive fusion based on RSSI. Data are collected from stationary sector sensors, mobile receivers, and UAV platforms, then undergo normalization and robust aggregation, after which they are combined using weighted fusion that accounts for channel quality, antenna characteristics, and noise. Coordinate estimation is then performed with the generation of confidence intervals (68% and 95%).

At the output, an SIEM event is generated containing the estimated coordinates, uncertainty parameters, a confidence score, and evidentiary telemetry (for audit purposes). Dashed lines indicate the permitted correlation only between monitor-mode observations and protected network data. IEEE 802.11mc FTM/RTT is marked as active-only and excluded from the passive methodology.

The antenna infrastructure model defines the baseline geometry and stability of the sensing layer, which directly affects posterior quality and distance-related error qualification. The infrastructure is divided into stationary and mobile components.

The stationary layer provides stable observation geometry and temporal consistency, while the mobile layer (vehicles/UAVs) increases observation diversity, reduces geometric degeneracy, and improves statistical accumulation. The fixed deployment parameters are listed in Table 1.

Thus, sector antennas provide controlled perimeter sensitivity and calibration stability, while omnidirectional antennas on mobile platforms provide spatial “saturation” of observations. The combination of these two layers minimizes geometric ambiguities and improves the robustness of Bayesian estimation under field conditions.

The localization pipeline and the blockchain evidentiary layer are coupled through a structured event record produced at each temporal step t. This coupling is bidirectional. First, localization confidence governs whether the smart contract authorizes an action. Second, the smart contract policy thresholds define the confidence levels that the calibration stage must satisfy before automated actions are permitted.

Downward coupling (localization to blockchain): At each step, the Bayesian pipeline produces the posterior $p(x_{t}|z_{1:t})$, the MAP estimate ${\hat{x}}_{t}$, the confidence region $R_{\alpha ,t}$, the calibrated confidence score $C_{t}$, and the SOC decision $D_{t}\in \{AUTO,VERIFY,OBSERVE\}$.

These outputs are serialized into a structured event record containing the hash of the raw telemetry, the estimated coordinates, the confidence score, the decision label, the confidence-region summary, the timestamp, the sensor identifiers, and the model version. The raw telemetry remains off chain in a protected evidence container, while the event record is submitted to the permissioned ledger.

Smart-contract gating: The smart contract authorizes automated action only when two conditions are satisfied: (i) the calibrated confidence score exceeds the policy threshold required for the requested action level, and (ii) the evidentiary record is complete and hash-verifiable. If either condition fails, the system is downgraded to VERIFY or OBSERVE mode, and the downgrade reason is recorded.

Upward coupling (blockchain to localization): The policy thresholds embedded in the smart contract define the confidence levels that the calibration stage must achieve before automated actions are permitted. When these thresholds are updated during periodic risk review, the updated values are propagated back to the localization calibration stage so that the statistical model and the operational policy remain synchronized.

Security threats addressed by the framework: At the radio layer, management-frame flooding can disrupt the localization pipeline through burst injection of spoofed beacon or probe frames [11]. This risk is mitigated in Stage 1 through robust median-based RSSI aggregation and temporal windowing. MAC address randomization complicates persistent source tracking over time; temporal filtering (Section 4.7) and frame-feature clustering partially compensate for this effect. Rogue access-point injection can also introduce false localization anchors [22,23]; to reduce this risk, the sensor-weighting scheme in Section 4.3 down-weights sensors exhibiting anomalous occupancy or noise behaviour.

At the transport and analytics layers, telemetry may be transmitted over standard protocols such as TCP and QUIC. However, transport-layer protection alone does not prevent modification by a privileged insider or a compromised analytics node after the data have crossed the transport layer [18]. The on-chain hash-anchoring mechanism addresses this limitation: once the structured event record is committed to the ledger, any post hoc modification of the localization result or its evidentiary fields becomes detectable through hash recomputation and comparison.

At the decision and audit layers, unauthorized modification of SOC response thresholds could silently lower the confidence bar for automated action. This risk is mitigated because the smart contract encodes the relevant policy thresholds in auditable on-chain logic, requiring a documented update path for any change. Post-incident repudiation is likewise reduced by the on-chain record, which preserves the confidence score, evidence hash, and policy label at the moment of decision. Importantly, the blockchain layer does not improve localization accuracy; its security value lies in tamper evidence, non-repudiation, and verifiable chain-of-custody for localization-derived decisions in high-stakes SOC environments.

The Methods section establishes a complete SOC-oriented pipeline for passive Wi-Fi localization in critical infrastructure environments, from telemetry collection to operational decision-making.

First, technological separation is enforced: passive radio data (RSSI, management/control frames, receiver logs) are separated from protected network events and SOC response logic. Allowed correlation is restricted only to monitor-mode observations and security logs/events, while IEEE 802.11mc FTM/RTT is explicitly classified as active-only and excluded from the passive methodology.

Second, the localization methodology is defined as probabilistic: the output is posterior uncertainty and confidence regions. This allows the system to manage operational risk through SOC decision thresholds (AUTO/VERIFY/OBSERVE) rather than rely on a single coordinate estimate.

Third, the approach incorporates robustness to outliers and interference, as well as temporal consistency for intermittent or moving sources, which is critical in real-world environments.

Fourth, the practical driver of localization quality is parameterized through large-scale mobile data collection and UAV-based sensing. In addition, the antenna infrastructure model is defined as a mandatory component of the method: stationary sector sensors ensure stable observation geometry and repeatability, while omnidirectional antennas on mobile platforms increase observation diversity and reduce geometric ambiguity.

Thus, the Methods section defines a reproducible framework specifying:

- What data are collected passively;
- How localization estimates with uncertainty are produced;
- How uncertainty is calibrated and converted into response thresholds;
- What infrastructure and field protocols ensure estimation quality.

The following Results section presents empirical findings from field validation, including error distributions and CDF analysis, demonstration of distance-related degradation with an error “spike” beyond approximately 40 m, examples of posterior maps and confidence regions, and the effect of uncertainty-oriented decision thresholds on the frequency of false tactical actions and SOC response latency.

## 7. Experimental Setup

Field validation is the primary approach.

The experimental protocol is based primarily on field data collection, conducted either by driving or flying around the monitored zone or by deploying sensors around the facility perimeter. Any synthetic or simulated data, if included, are used only as auxiliary support and are explicitly identified as secondary to the field validation process.

### 7.1. Field Campaign Plan (2–3 Days)

A field campaign lasting 2–3 days provides a sufficient volume of data for statistical analysis. The plan includes:

Stationary layer: sector sensors deployed at key perimeter points (Table 1).
Mobile layer: a vehicle equipped with an omnidirectional antenna following routes from multiple viewing angles to increase geometric diversity.
UAV layer: flight along predefined routes with a measurement rate of approximately 1 Hz to accumulate statistical observations.

### 7.2. UAV Collection Parameters

The unmanned aerial platform is used as a standardized mobile data collector to increase geometric diversity of observations and enable statistical accumulation of measurements. Unlike the stationary layer, UAVs allow controlled variation in altitude, observation angle, and trajectory, which directly reduces posterior ambiguities.

Main collection parameters:

Altitude ~50 m: capture radius up to approximately 0.8–1.0 km in open environments.
Altitude ~100 m: capture radius up to approximately 1.5 km from the flight path (in the absence of significant shielding).
Measurement frequency: 1 measurement per second (1 Hz) with subsequent robust aggregation within temporal windows.

A 1 Hz sampling rate provides sufficient statistical accumulation without overloading the channel and enables the formation of stable RSSI aggregates for subsequent Bayesian fusion.

The altitude mode is selected depending on the built environment configuration. In areas with partial or heavy NLOS conditions, increasing altitude reduces shielding effects but increases attenuation variability; this effect is accounted for in the range-dependent uncertainty model.

**Figure 5:**
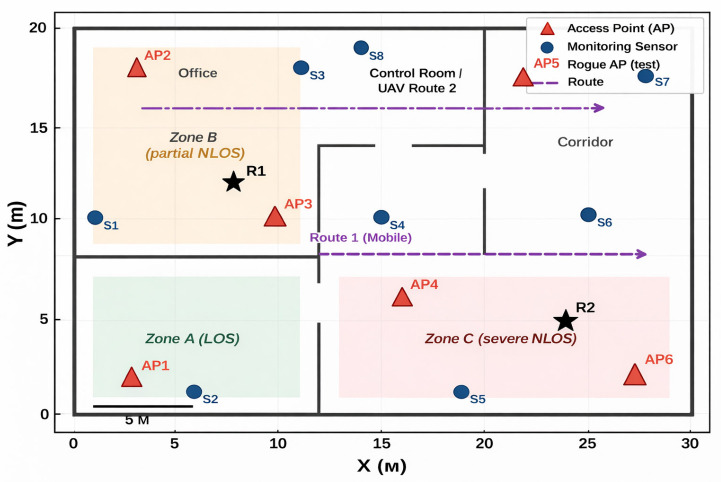
Field experiment plan: placement of stationary sector sensors and trajectories of mobile/UAV data collection using an omnidirectional antenna. The star indicates the monitored critical infrastructure facility/reference point.

Figure 5 illustrates the experimental plan, including the placement of stationary sector sensors and the trajectories of mobile and UAV-based data collection using an omnidirectional antenna. The axes X(m) and Y(m) represent the easting and northing distances, respectively, in metres within a local planar coordinate system centred on the facility perimeter reference point. Zones labelled LOS, partial NLOS, and severe NLOS represent different signal-propagation regimes used for range-based error qualification and for validating the localization error spike beyond approximately 40 m. Flight trajectories are designed to intersect different geometric configurations of sensors to minimize solution degeneracy.

### 7.3. Evaluation Metrics and Range Qualification

The evaluation consists of two components: localization quality considering uncertainty, and the quality of SOC thresholds as a function of calibrated confidence.

Localization Accuracy and Range Qualification

Localization error:

(14) $$e_{t}=∥{\hat{x}}_{t}-x_{t}^{*}{∥}_{2}$$

where ${∥\cdot ∥}_{2}$ denotes the Euclidean (L_2_) norm, i.e., the straight-line distance in the plane between the estimated coordinates ${\hat{x}}_{t}$ = [${\hat{x}}_{t,1}$, ${\hat{x}}_{t,2}$]^T^ and the reference (ground-truth) coordinates xt* = [$x_{t,1}^{*}$, $x_{t,2}^{*}$]^T^, with units of metres.

The analysis is performed separately for two distance regimes, namely d≤40 m and d>40 m, with explicit demonstration of the degradation spike observed beyond the operational validity boundary.

Supporting materials for this study, including visualizations, explanatory captions, and comments for Figures S1–S8, are available in the Zenodo repository under DOI: 10.5281/zenodo.18809252.

## 8. Results

The empirical results are presented through four complementary visual summaries: localization-error CDFs, ROC curves for SOC threshold selection, posterior heatmaps with confidence ellipses, and a range-regime comparison separating the validated near field (≤40 m) from the degraded far field (>40 m). The main empirical findings and their operational implications are summarized in Table 2.

### 8.1. Localization Error Distribution

Localization performance was evaluated using the cumulative distribution function (CDF) of localization error, with particular attention to the upper tail because large errors are the most relevant for operational decision-making. The near-range regime remains comparatively stable, whereas the far-range regime exhibits a heavier error tail. This behavior supports the use of distance-qualified response thresholds rather than a single policy across the entire monitored area. For SOC operations, average localization error alone is insufficient: the tails of the error distribution are critical, because they create the risk of incorrect tactical actions. Therefore, localization quality is evaluated using the error CDF and range-based qualification of result validity (before/after a threshold).

The error CDF therefore provides an operationally meaningful summary: the near-range tail is dominated by small errors suitable for AUTO playbooks, while the far-range tail contains errors large enough to trigger false or disruptive automated actions—establishing the practical case for range-qualified SOC thresholds.

**Figure 6:**
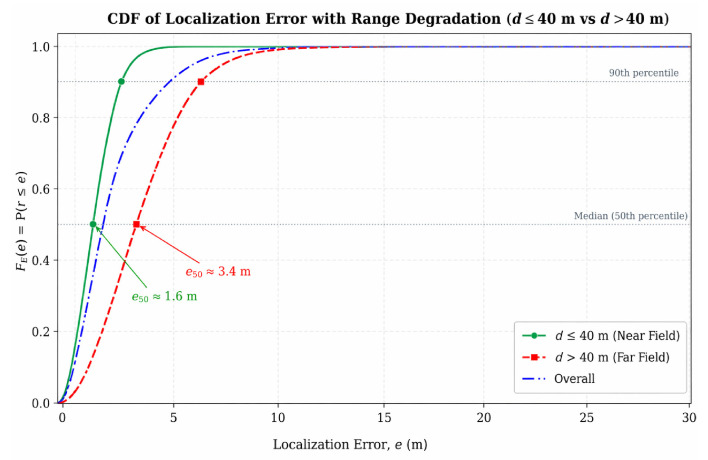
Range-dependent localization degradation under interference and shielding.

In the valid range (up to ≈40 m), the curve rises more rapidly, indicating a larger proportion of small localization errors. In contrast, Figure 6 shows that beyond ≈40 m a clear error spike emerges together with a heavier tail, indicating pronounced degradation in localization quality under hostile or challenging influences such as interference and shielding. This breakpoint is therefore treated as a practical boundary for range-based accuracy qualification and for more conservative SOC threshold tuning.

Beyond approximately 40 m, the localization regime shifts from one in which AUTO playbooks may be appropriate to one in which VERIFY mode or additional data collection should be required before any automated containment action is authorized. The more gradual rise in the far-field CDF in Figure 6 further confirms that uncertainty increases substantially once the operational validity boundary is exceeded.

### 8.2. Threshold Quality for SOC Actions (ROC)

ROC analysis was used to evaluate the trade-off between detection sensitivity and false actions when setting AUTO and VERIFY thresholds. As expected, the more conservative AUTO threshold reduces false positives, while the VERIFY threshold accepts a higher false-positive rate in exchange for increased sensitivity. These curves provide a direct basis for selecting response policies according to the operational cost of error.

The ROC curves thus provide a formal, operationally interpretable basis for selecting response policies: the AUTO operating point minimizes false-positive automated actions at the cost of lower detection sensitivity, while the VERIFY operating point accepts a higher false-positive rate in exchange for greater coverage—a trade-off that should be calibrated to the cost-of-error profile of the specific critical infrastructure environment.

ROC curves describe SOC decision thresholds in the AUTO versus VERIFY scenario. AUTO uses a higher threshold, resulting in fewer false actions, whereas VERIFY uses a lower or moderate threshold, yielding more detections at the cost of a higher false-positive rate (FPR). The marked operating points indicate specific threshold configurations, and the area under the curve (AUC) serves as an overall measure of threshold quality. In practical terms, ROC analysis enables the formal selection of thresholds based on the cost of error in critical infrastructure environments: for destructive actions, minimizing false positives is the priority, whereas softer actions or verification workflows may tolerate more triggers with a controlled FPR.

### 8.3. Posterior Visualization and Confidence Regions

The visualization shows a heatmap of the posterior density together with the MAP estimate and the 68% and 95% confidence ellipses. This representation is more informative than a point estimate alone because it conveys both the most likely location and the spatial extent of uncertainty relevant to triage and playbook selection. In the SOC context, it is essential to represent results as a probability distribution with confidence regions rather than as a single coordinate point, because decisions depend not only on location but also on the size and placement of the uncertainty region.

The confidence ellipses can therefore be interpreted as a spatial risk budget: a compact 68% ellipse concentrated in an accessible zone may support an AUTO response, whereas a large or irregularly shaped 95% ellipse spanning multiple zones argues for VERIFY or OBSERVE. The brightest region in the posterior map corresponds to the most probable location, while the ellipses represent compact and extended areas of possible coordinates. The key conclusion is that localization becomes operationally controllable: instead of receiving only a point estimate, the SOC receives a coordinate hypothesis, its spatial uncertainty, and an explicit basis for triage decisions.

### 8.4. Error Spike Beyond ~40 m (Field Effect)

Field data indicate a pronounced degradation in localization performance beyond approximately 40 m. In this regime, the error distribution shifts toward larger values, and the uncertainty region expands, rendering coordinate-based automation less reliable. This breakpoint is therefore interpreted as an operational validity boundary rather than merely as a descriptive characteristic of the data.

From an operational perspective, beyond 40 m the priority shifts from maximizing automated responses to minimizing erroneous actions. Accordingly, OBSERVE mode and supplementary mobile or UAV-assisted data collection are preferred to reduce positional uncertainty before any automated playbook is activated.

To reflect this transition, the error CDF is analyzed separately for two distance regimes: ≤40 m and >40 m. In the near zone, the curve rises rapidly, indicating more stable localization, whereas in the far zone it shifts rightward and exhibits a pronounced degradation spike with a heavier tail. These results support range-based accuracy qualification and more conservative SOC thresholds in high-uncertainty areas. Practically, this means that the same localization model should not yield equally confident conclusions at all distances; in the far zone, the priority is to minimize false actions rather than maximize automation.

### 8.5. Operational Integration (SIEM–SOAR) and Evidentiary Auditability

For practical deployment, localization results and calibrated confidence scores must be exported to SIEM and used to govern SOAR playbook execution through the AUTO, VERIFY, and OBSERVE decision modes.

The pre-association indicators extracted from management frames serve a dual role: they contribute directly to the posterior spatial estimate and, once exported to SIEM together with their evidentiary hash, provide an independently verifiable record that a given Wi-Fi event was observed and localized before any SOC action was taken.

## 9. Discussion

Interpretation of Results and SOC Implications

The main practical finding is that, in SOC settings, localization quality should be assessed not only by central tendency but also by the tail of the error distribution. Large errors are operationally more consequential than small average gains in nominal accuracy because they are the cases most likely to trigger an incorrect response. In that sense, uncertainty calibration is not an auxiliary output of the model; it is part of the decision logic.

Comparison with Other Approaches and SOC Practice

In most applied localization solutions, results are presented as point estimates, which are convenient for visualization in user interfaces but fragile under real-world conditions. Multipath propagation, shielding, interference, and environmental variability make a single “best” coordinate potentially unsafe for automated response systems.

In contrast, the proposed approach—based on posterior coordinate distributions and confidence regions—aligns localization outputs with the risk-oriented thinking typical of SOC operations. Decisions are therefore triggered not by the existence of a coordinate estimate itself, but by the presence of sufficient calibrated confidence and an acceptable risk of error.

Compared with approaches that emphasize high localization accuracy through active procedures, this work intentionally relies exclusively on passive monitor-mode data. While this may reduce best-case instrumental accuracy, it significantly improves realism for SOC deployments, where active client participation is often impossible or undesirable. It also simplifies acceptable correlation policies within security monitoring frameworks.

Interpretation of the Error “Spike” Beyond ≈ 40 m as an Operational Validity Boundary

The degradation observed beyond approximately 40 m is best interpreted as a shift in the underlying observation regime. At longer distances, non-line-of-sight propagation, attenuation variability, local reflections, and interference become more influential, reducing the reliability of passive measurements. From an operational perspective, this argues against treating all coordinates equally. Beyond that range, the system should prioritize verification or additional data acquisition rather than direct automated action.

The practical implication is that, beyond this boundary, localization should transition from reporting coordinates to reporting uncertainty and recommending actions. Accordingly, in regions beyond 40 m, automatic SOC playbooks should be significantly restricted, while VERIFY actions or additional mobile/UAV-based data collection should be prioritized to reduce uncertainty.

Confidence Calibration as a Bridge between Statistics and Tactical Decision-Making

In this framework, calibration metrics (empirical coverage and reliability diagrams) function as a translator between mathematical uncertainty and operational risk management. The same numerical confidence value, if not calibrated, may correspond to very different real-world risks depending on the environment.

Therefore, SOC thresholds must rely on calibrated confidence rather than raw probability estimates; otherwise, automation becomes effectively uncontrolled. In this sense, ROC-based tuning of AUTO/VERIFY thresholds is not merely a model evaluation metric but a practical mechanism for formally adjusting response policies according to the cost of error within a given critical infrastructure environment.

Study Limitations

Noise in passive features: RSSI measurements and frame patterns observed in monitor mode strongly depend on channel conditions, hardware differences between receivers, client power-saving modes, and external interference. This necessitates robust estimation mechanisms and careful interpretation of results, particularly at longer ranges.

Domain shift: Seasonal or infrastructural changes (e.g., building modifications, new interference sources, or equipment relocation) may alter feature statistics and degrade calibration quality. In practice, this implies the need for periodic recalibration and continuous monitoring of model performance.

MAC randomization and intermittency: In some scenarios, source tracking over time is complicated by randomized device identifiers and short bursts of activity. Temporal filtering can mitigate this issue but cannot guarantee continuous tracking.

Infrastructure requirements: A stationary localization framework using sector antennas requires periodic calibration. Without such calibration, the risk of systematic localization bias increases.

Practical Implications for Zero Trust and Multimodal AI

Within a Zero Trust perspective, spatial attribution should be treated as evidence with associated confidence rather than as a deterministic fact. Destructive or high-impact actions should therefore depend on calibrated uncertainty estimates that are consistent with the organization’s risk tolerance. This principle fits naturally within SIEM–SOAR workflows that already rely on structured conditions for escalation and automation.

It also aligns naturally with SIEM–SOAR ecosystems, where events can include uncertainty fields and evidentiary attributes that support auditing and reproducibility of response actions.

A promising direction for further development is multimodal AI, combining radio features (RSSI and frame patterns) with network security events (NAC, IDS, EDR), contextual access information (time, location, policy), and—when permitted—additional sensor channels such as video surveillance operating at the level of zone presence rather than personal identification. Such integration could reduce uncertainty and improve playbook gating decisions.

At the same time, it remains essential to maintain data minimization principles and strict correlation controls, ensuring that the system does not evolve into an opaque and difficult-to-audit data aggregation platform.

Reproducibility and Open Materials

The materials supporting the visualizations and textual interpretation of the results have been published in an open Zenodo repository. This improves the verifiability of the conclusions and enables practitioners to adapt thresholds and policies to specific critical infrastructure environments without altering the fundamental methodological principles.

The blockchain extension should be interpreted as an evidentiary-control mechanism rather than as a claim that distributed ledgers improve localization accuracy. Its value lies in strengthening integrity, provenance, and non-repudiation of localization-related decisions. For critical infrastructure SOCs, this is especially relevant when posterior estimates trigger operationally consequential actions or when incident reconstruction must establish which evidence was available at decision time. A permissioned, hybrid on-chain/off-chain design appears to be the most realistic option because it preserves the confidentiality of raw telemetry while enabling integrity proofs and response-policy transitions to be independently verified [2,3,6].

## 10. Conclusions

This study shows that passive Wi-Fi localization for critical infrastructure SOCs is more effective when expressed as a posterior spatial estimate with calibrated uncertainty rather than as a single coordinate. The proposed Bayesian framework connects uncertainty estimation to operational response control through the AUTO, VERIFY, and OBSERVE modes and extends localization events with permissioned evidentiary logging to improve auditability.

Field validation indicates a marked degradation in localization performance beyond approximately 40 m, which should be regarded as a practical boundary for confident automated action. Within the validated range, calibrated confidence supports more defensible response decisions; beyond that range, additional observation or verification is preferable. In this context, the blockchain layer should be understood as an evidentiary safeguard that preserves integrity and provenance rather than as a means of improving localization accuracy.

The results further show that calibrated confidence can serve as an effective control parameter for tuning response policies. A conservative AUTO threshold reduces the risk of erroneous high-impact actions, whereas the VERIFY threshold supports incident triage with an acceptable increase in verification workload. Representing localization outputs as posterior distributions with confidence regions therefore provides the SOC with both a location estimate and an explicit basis for judging whether that estimate is sufficiently reliable to justify action.

Overall, this study defines an SOC-compatible pipeline that combines passive monitor-mode observations, Bayesian localization, uncertainty calibration, decision-threshold control, and export of evidentiary telemetry to SIEM/SOAR.

Future work should focus on adaptive threshold learning, robust calibration under domain shift, optimized mobile and UAV-assisted collection trajectories, and standardized SIEM schemas for uncertainty-aware, audit-ready evidentiary records [24,25].

## Figures and Tables

**Table 1:** The antenna infrastructure model.

Antenna Type	Deployment	Platform	Notes
Omnidirectional	Mobile deployment	Mobile platform (vehicle, UAV)	Used for in-motion data collection; provides 360° coverage.
Sector	Stationary deployment	Critical infrastructure facility	Fixed installation; calibration at least four times per year.

**Table 2:** Summary of principal empirical findings and operational implications.

Result Category	Main Empirical Finding	Operational Implication
Localization uncertainty	Posterior distributions provide more informative outputs than point estimates alone	SOC actions should be based on confidence-aware localization rather than on single coordinates
Range effect	Localization performance degrades markedly beyond approximately 40 m	Fully automated action should be restricted beyond the validated range
ROC threshold tuning	AUTO reduces false actions, whereas VERIFY increases sensitivity at the cost of a higher false-positive rate (FPR)	Thresholds should be selected according to the operational cost of error
Posterior confidence regions	Large or dispersed confidence ellipses indicate spatial ambiguity	VERIFY or OBSERVE modes should be preferred when uncertainty regions are broad
Evidentiary logging	Permissioned blockchain strengthens integrity and provenance of localization records	Auditability and non-repudiation improve without placing raw telemetry on chain

## Data Availability

Supporting materials, including visualizations, field data descriptions, and result commentaries, are available in the Zenodo repository at DOI: 10.5281/zenodo.18809252.

## Supplementary Materials

Figures S1–S8 with detailed captions, field experiment result data, and operational interpretation of uncertainty metrics are available in the Zenodo repository at https://doi.org/10.5281/zenodo.19915303 (accessed on 25 April 2026).

## Abbreviations

The following abbreviations are used in this manuscript:

AP	Access Point
CDF	Cumulative Distribution Function
CII	Critical Infrastructure
FTM/RTT	Fine Timing Measurement/Round-Trip Time (IEEE 802.11mc; active-only)
MAC	Media Access Control address (subject to randomization)
NLOS	Non-Line-of-Sight
ROC	Receiver Operating Characteristic
RSSI	Received Signal Strength Indicator
SIEM	Security Information and Event Management
SOAR	Security Orchestration, Automation and Response
SOC	Security Operations Center
STA	Station (client device)
UAV	Unmanned Aerial Vehicle
Mathematical Notation	
Symbol/Notation	Description
x ∈ ℝ^2^	$\mathrm{Source}\;\mathrm{coordinates}\;\mathrm{in}\;\mathrm{the}\;\mathrm{plane},\;x=[x_{1},x_{2}{]}^{T}$
t	Discrete time
Δt	Discretization step
s_i_ ∈ ℝ^2^	Coordinates of sensor i, i∈1,…,N
z_i,t_	Passive observation of sensor i at time t (RSSI statistics, management/control frame features, logs)
z_t_ = {z_i,t_}_i__=__1_^N^	Set of observations at time t
p(z|x)	Likelihood
p(x|z)	Posterior distribution
$\hat{x}$	Point estimate of the source location, obtained for example as the maximum a posteriori (MAP) estimate or posterior mean derived from p(x∣z)
Σ	Posterior covariance (Gaussian approximation)
Rα	Confidence region at level α (ellipse/HPD)
d_i_(x) = ‖x − s_i_‖_2_	Distance from sensor i to point x
w_i_	Sensor weight coefficient (reliability, geometry, channel occupancy)
